# Cytotoxicity of CD19-CAR-NK92 cells is primarily mediated via perforin/granzyme pathway

**DOI:** 10.1007/s00262-023-03443-1

**Published:** 2023-04-13

**Authors:** Jonas Althaus, Verena Nilius-Eliliwi, Abdelouahid Maghnouj, Sascha Döring, Roland Schroers, Michael Hudecek, Stephan A. Hahn, Thomas Mika

**Affiliations:** 1 Department of Molecular Gastrointestinal Oncology, Ruhr University Bochum, Bochum, Germany; 2 Department of Medicine, Hematology and Oncology, Knappschaftskrankenhaus Bochum, Ruhr University Bochum, In der Schornau 23-25, D-44892 Bochum, Germany; 3 Department of Internal Medicine 2, University Hospital of Würzburg, Würzburg, Germany

**Keywords:** Chimeric antigen receptor, CAR, Lymphoid malignancies, NK92-cells, Perforin, Granzyme, Death receptor

## Abstract

Chimeric antigen receptors (CARs) have improved cancer immunotherapy in recent years. Immune cells, such as Natural killer cells (NK-cells) or T cells, are used as effector cells in CAR-therapy. NK92-cells, a cell line with known cytotoxic activity, are of particular interest in CAR-therapy since culturing conditions are simple and anti-tumor efficacy combined with a manageable safety profile was proven in clinical trials. The major pathways of immune effector cells, including NK92-cells, to mediate cytotoxicity, are the perforin/granzyme and the death-receptor pathway. Detailed knowledge of CAR-effector cells’ cytotoxic mechanisms is essential to unravel resistance mechanisms, which potentially arise by resistance against apoptosis-inducing signaling. Since mutations in apoptosis pathways are frequent in lymphoma, the impact on CAR-mediated cytotoxicity is of clinical interest. In this study, knockout models of CD19-CAR-NK92 cells were designed, to investigate cytotoxic pathways in vitro*.* Knockout of *perforin 1* (*Prf1*) and subsequent abrogation of the perforin/granzyme pathway dramatically reduced the cytotoxicity of CD19-CAR-NK92 cells. In contrast, knockout of *FasL* and inhibition of TRAIL (tumor necrosis factor-related apoptosis-inducing ligands) did not impair cytotoxicity in most conditions. In conclusion, these results indicate the perforin/granzyme pathway as the major pathway to mediate cytotoxicity in CD19-CAR-NK92 cells.

**Supplementary Information:**

The online version contains supplementary material available at 10.1007/s00262-023-03443-1.

## Introduction

Cancer immunotherapy has become an important tool for cancer treatment in recent years. By the introduction of chimeric antigen receptors (CAR), stably transduced into immune effector cells, gene therapy and cancer immunotherapy have been successfully combined. The first studies of immune effector cells and CARs focused on T cells, genetically altered by lentiviral or retroviral gene transfers, targeting cancer cells [1, 2]. Pursuing this approach, different CAR-T cell products are in clinical use [3–6].

Despite remarkable response rates in heavily pretreated patients, different factors limit the utility of CAR immunotherapy. Cytokine release syndrome (CRS) and immune effector cell-associated neurologic syndrome (ICANS) are potentially life-threating adverse events of CAR-T cell therapy [7]. The risk of HLA-mediated graft-versus-host (GvH) reactivity restricts CAR-T cell therapy mainly to autologous use. Preceding chemotherapy, T cell exhaustion and an immunosuppressive tumor microenvironment are host factors, further restraining CAR-T cell therapy [8–10]. Thus, further improvement in CAR-based immunotherapy is required.

NK-cells represent a potent alternative as CAR-effector cells, overcoming important disadvantages of T cells [11, 12]. NK-cells can be used as an allogeneic off-the-shelf product, because full HLA matching is unnecessary. A favorable cytokine/chemokine profile appears to be accompanied by a lower risk of CRS and ICANS [11, 13]. NK-cells can be conveniently isolated from peripheral blood and umbilical cord blood of human donors. In an early clinical trial, allogeneic CAR-NK cells obtained from umbilical cord blood have shown high efficacy and a remarkable safety profile [14]. In addition, irradiated cell lines such as NK92-cells can be applied safely [11, 12, 14, 15]. The NK92 cell line is of particular interest, since culturing conditions are simple and preclinical as well as clinical trials proved their cytotoxic activity [16–18].

Cytotoxicity of NK-cells is mainly mediated via degranulation of perforin and granzymes and by expression of death-ligands (FasL and TRAIL) [19]. Binding to their corresponding receptor on the target cell, these ligands induce apoptosis by activating the death-receptor pathway [19, 20]. Despite these effective mechanisms, CAR-therapy may be unsuccessful, because of target cells’ resistance towards either pathway [21].

Downstream signaling after CAR activation and subsequent cytotoxic action is under current investigation and partly unknown [22–24]. Understanding the cytotoxic mechanisms of CAR-effector cells is essential to unravel and may overcome resistance of target cells. It is unclear if the perforin/granzyme pathway and the death ligand pathways can be substituted by each other, or if the loss of one pathway, for example by acquired resistance mechanisms in the target cells, leads to a substantial decrease in cytotoxic activity in CAR-NK92 cells.

In this study, we performed in vitro experiments with different CD19-CAR-NK92 knockout (ko) models to provide further insight into cytotoxic pathways of these cells.

## Materials and methods

### Cell lines and human primary cells

NK92-cells were used as CAR-effector cells. Daudi and Karpas422 cells (CD19^+^ non-Hodgkin lymphoma cell lines), human primary B cells and Jurkat cells (CD19^−^) served as target cells. All cell lines were authenticated by STR analysis.

NK92-cells were cultured in RPMI1640 (GIBCO) supplemented with 10% FBS (GIBCO), 4 mM L-glutamine (GIBCO), 100 U/ml/100 µg/ml penicillin/streptomycin (Sigma-Aldrich), 10 mM HEPES (Sigma-Aldrich) and 100 U/ml recombinant human Interleukin (IL)-2 (Novartis). Other cell lines were cultured in RPMI1640, containing 2 mM L-glutamine and 50 U/ml/50 µg/ml penicillin/streptomycin supplemented with 20% FBS for Karpas422, 10% FBS for Jurkat or 10% FBS, 1 mM sodium pyruvate (GIBCO) and 10 mM HEPES for Daudi.

#### Collection and culture of primary B cells

The ethical committee of the Ruhr-University Bochum approved the collection and experimental use of human blood samples (No. 18-6462). All donors gave their informed consent and participated voluntarily. First, peripheral blood mononuclear cells (PBMCs) were isolated by density gradient centrifugation, as previously described [8].

The isolation of primary B cells from human PBMCs was performed using the Pan B cell Isolation Kit (human), MidiMACS™ Separator and LS Columns (all Miltenyi Biotec) according to the manufacturer’s instructions. The purified cell suspension was centrifugated at 400 × g for 5 min and cultured in B cell growth medium (RPMI 1640) supplemented with 10% FBS, 4 mM L-glutamine, 100 U/ml/100 µg/ml penicillin/streptomycin, 10 mM HEPES, 1 mM sodium pyruvate, 200 U/ml IL-2, 10 ng/ml IL-4 and 10 ng/ml BAFF (both Peprotech). B cells were cultured for 24 to 48 h until application in further experiments.

### Gene editing of effector and target cells

Different CD19-CAR-NK92 knockout models were designed using a CRISPR-Cas9 system. The target-cell lines (Daudi, Karpas422, and Jurkat) were designed as green fluorescent protein (GFP) expressing cells, as described below. The plasmids used comprised antibiotic selectable markers and were designed according to following strategy: Plasmids were digested with appropriate restriction enzymes (all NewEngland Biolabs). The intentional DNA-fragments were separated by gel-electrophoresis and extracted using the FastGene Gel/PCR Extraction kit (Nippon Genetics). The required inserts and vectors were ligated by T4-DNA-Ligase (Invitrogen).

#### 4-1BB anti-CD19 CAR construct

We used a second-generation anti-CD19 CAR, harboring the FMC63 antigen binding domain and a 4-1BB co-stimulatory domain (plasmid pJ2459_19shBB, a kind gift of Michael Hudecek, University of Würzburg, Germany) [25, 26]. The CAR-sequence was cloned into the pLJM1-eGFP-Puro (#19319, Addgene) instead of the *eGFP* gene (restriction-enzymes: NheI (5’-end) and EcoRI (3’-end)).

#### Lentiguide-sgRNA-Prf1-ko-Neo, lentiguide-sgRNA-FasL-ko-Neo, and empty vector

GuideRNA-coding vectors harbored the specific sequences for *Prf1*-ko (*Prf1*-ko: sense 5’-CACCGGAGGAGCAGACGGGCTGCC-3’) and *FasL-ko (FasL*-ko: sense 5’-CACCGGTAATTGAAGGGCTGCTGCA-3’), respectively. Each sequence was inserted into the BsmBI-restricted lentiGuide-Puro (#52963, Addgene). The puromycin resistance gene of both resulting plasmids was replaced by the neomycin resistance gene of LentiGuide-Neo (#139449, Addgene;: XmaI (5’-end) and MluI (3’-end)). An empty LentiGuide-Neo was designed by removing the stuffer sequence from original LentiGuide-Neo (#139449, Addgene).

#### Lenti-eGFP-blast

The GFP-coding DNA-sequence of pLJM1-eGFP-Puro (#19319, Addgene) was PCR-amplified (Primers: sense 5’-CGCTACCGGTGCGCCGGACACGCTGAAC-3’; antisense 3’-CGCTGGATCCCTTGTACAGCTCGTCCATG-5’) and cloned into lentiCas9-Blast (#52962, Addgene) instead of the *Cas9*-sequence [AgeI (5’-end) and BamHI (3’-end)].

### Lentiviral transduction

Lentiviral vector production was carried out as previously described [8, 27]. Transduction of target lymphocytes was carried out for 24 h applying the lentiviral supernatant supplemented with 8 µg/ml polybrene (Sigma-Aldrich).

To enhance the transduction of NK92, Daudi, and Karpas422 cells, transduction was carried out in the presence of the TBK1/IKK inhibitor BX795 (8 µM, Sigma-Aldrich) for 5 h, as previously described. [28]. Afterwards, the medium was replaced with fresh viral supernatant for continuing 19 h, supplemented only with polybrene. After transduction, the cells were washed and cultured in the cell line specific growth medium.

NK92-cells were sequentially transduced, first with the anti-CD19 CAR and then with the plasmids for CRISPR/Cas9-mediated knockouts. Prior to any transduction, NK92-cells were activated with 1000 U/ml IL-2 for 16 h, as previously described [28]. During transduction, the IL-2 concentration was kept at 100 U/ml. The target cells (Jurkat, Karpas422, and Daudi) were transduced with the GFP-coding lentivector.

Transduced cells’ selection was started three days after transduction and was maintained for the entire cell culture period. For NK92-cells, puromycin (Sigma-Aldrich) was used at 2 µg/ml, blasticidin (Roth) at 9 µg/ml and G418 (GIBCO) at 0.5 mg/ml. For Daudi cells, blasticidin was used at 10 µg/ml and for Karpas422 cells at 8.5 µg/ml.

### CRISPR/Cas9-mediated knockouts and creation of NK92 cell clones

After transducing LentiCas9-Blast and following blasticidin selection, NK92 cells were transduced with either LentiGuide-sgRNA-*FasL*-ko-Neo (*FasL*-ko) or LentiGuide-sgRNA-*Prf1*-ko-Neo (*Prf1*-ko) or empty lentiGuide-Neo (reference). After subsequent neomycin selection, a proportion of NK92-*FasL*-ko cells were transduced with the LentiGuide-sgRNA-*Prf1*-ko-Neo to generate double-ko (*Prf1-*and *FasL*-ko) cell clones. The cells were kept under neomycin and blasticidin selection for at least 4 weeks before the single-cell clones were generated. For this purpose, a highly diluted cell suspension was seeded on 96-well plates with the aim of preserving 0.5–1 cell per well. Every 3–4 days fresh growth medium supplemented with IL-2 was added.

### Flow cytometry

CD19-CAR expression was analyzed using the CD19 CAR Detection Reagent (human, Biotin) and anti-biotin antibody (APC, both Miltenyi Biotec) according to the manufacturer´s protocol. To evaluate target cells’ CD19, FasR, TRAIL-R1 and -R2 expression, anti-CD19 (APC-Vio 770), anti-CD95 (APC), anti-CD261 (PE) and anti-CD262 antibodies (APC) were used (all Miltenyi Biotec). Analysis was performed using FACSCantoII (BD), and data analysis was carried out with FlowJo v10.6.1.

### Recombinant human FasL and cell viability assay (XTT-assay)

Target cells were incubated with recombinant human FasL (Cell Signaling Technology) in presence of 10 µg/ml anti-His antibody (R&D Systems) for 24 h. Cells were seeded on a 96-well plate in 100 µl medium at a cell count of 30,000 cells/well in case of the cell lines and 100,000 cells/well in case of primary B cells. Cell viability after treatment was assessed using the Colorimetric Cell Viability Kit III (XTT; PromoKine) according to the manufacturer’s protocol and measurement of the OD_450_–OD_630_.

### Western blots

Protein extraction and western blotting was carried out as previously reported [29]. Protein amounts were 28–30 µg per lane. Each sample was measured using the Pierce™ BCA Protein Assay Kit (Thermo Fisher Scientific). For protein detection, anti-perforin, anti-FasL, anti-TRAIL, and anti-*α* tubulin antibodies (all Cell Signaling Technology) were used.

### Cytotoxicity assays

Cytotoxicity was analyzed by flow cytometric detection of GFP^+^ or labeled (FITC^+^) target cells, as previously reported [30]. Jurkat, Karpas422, and Daudi cells were designed as GFP^+^ target cells, primary B cells were stained with anti-CD20 antibody (FITC, Beckman Coulter) immediately before FACS analysis. Target cells’ basal percentage of GFP^+^/FITC^+^ cells were analyzed for subsequent calculation of cell lysis. Effector (GFP^−^/FITC^−^) and target cells (GFP^+^ or FITC^+^) were incubated together for 4 h at various E:T ratios (1:4, 1:1, or 4:1) in 200 µl medium on 96-well plates. Cell count was 250.000 cells/well in all conditions. After co-incubation, the cell mixtures were analyzed regarding the respective proportion of GFP^+^ or FITC^+^ cells. To exclude cell debris and dead cells from analysis, viable cells were gated using forward and side scatter signals. Specific target-cell lysis was calculated as follows: (1-experimental GFP^+^/FITC^+^ proportion/starting GFP^+^/FITC^+^ proportion) * 100%.

#### Inhibition of TRAIL

Functional Grade CD253 (TRAIL) monoclonal antibody (RIK-2; eBioscience™; ThermoFischer) was used for TRAIL inhibition in cytotoxicity assays, as reported [31]. The effector cells were incubated with the anti-TRAIL antibody (1 µg in 100 µl cell suspension) for 1 h before they were mixed with target cells. During co-incubation, antibody concentration was adjusted to 5 μg/ml.

### Statistical analysis and graphical display

All statistical analyses and data plots were carried out with GraphPad Prism software (version 5.03). Unless stated otherwise, data values are expressed as means of multiple determination ± standard error of mean (SEM). Significance was tested by unpaired, two-tailed *t* tests or one-way ANOVA followed by Tukey’s multiple comparisons test. *P* values below 0.05 were considered to be significant and specified in gradations: ***, *p* < 0.001; **, *p* < 0.01; *, *p* < 0.05; ns, not significant.

## Results

### The 4-1BB CD19-CAR enhances cytotoxic activity

**Fig. 1:**
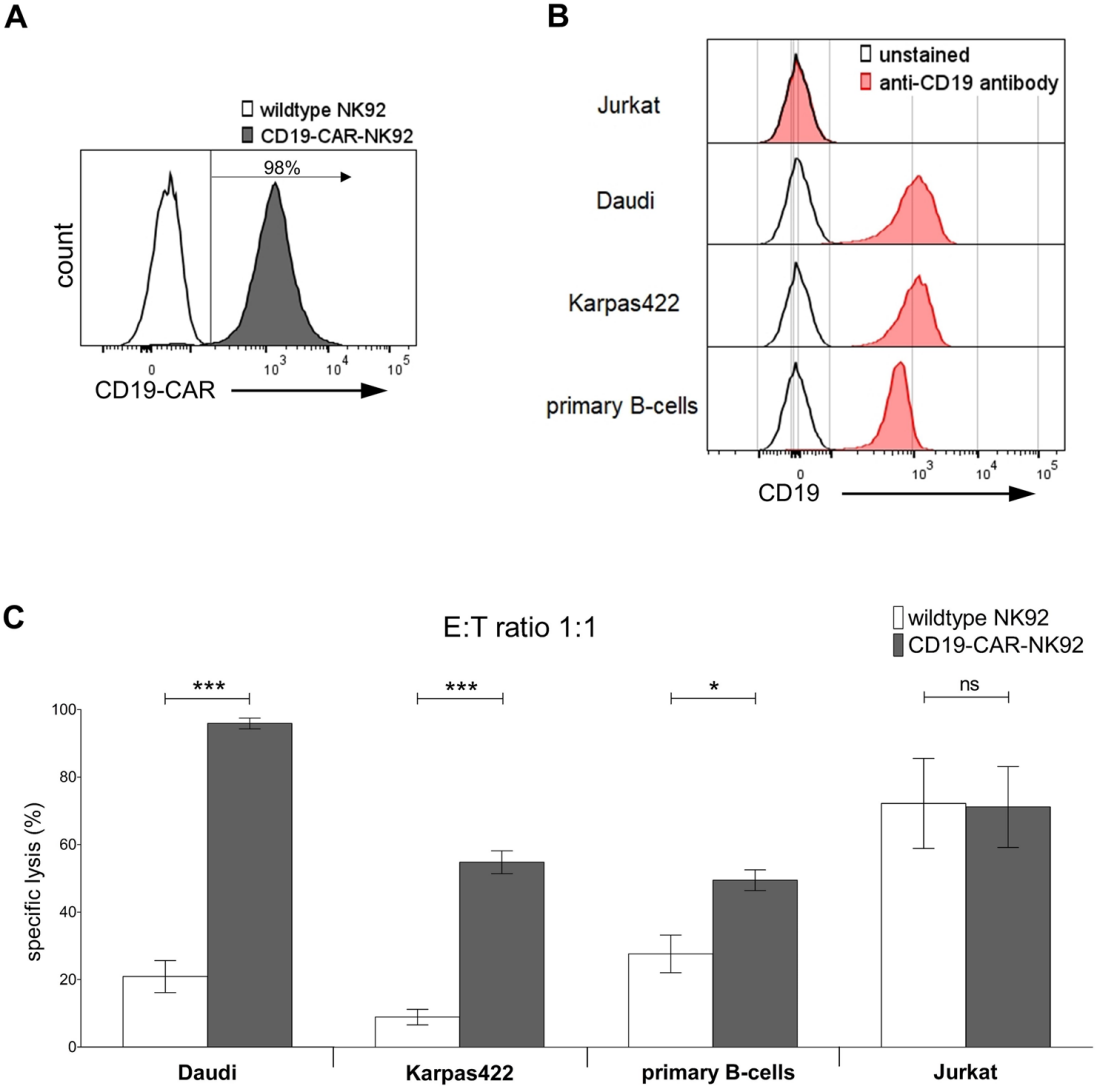
Improved cytotoxicity of CD19-CAR-NK92 cells versus CD19^+^ target cells **A** Histogram of effector cells’ CD19-CAR expression. **B** Histograms of target cells’ CD19 expression. **C** Specific lysis (%) of various CD19^+^ and CD19^−^ target cells by wildtype and CD19-CAR-NK92 cells at E:T ratio of 1:1 after co-incubation for 4 h. Each experiment was repeated 4 times per cell line (*n* = 4) and 2 times with primary B cells of 4 different healthy donors. Values are expressed as means of multiple determination ± SEM. *P* values were calculated by unpaired *t* test

Insertion of CARs into effector cells potentially overcomes immunoescape in malignant diseases and enhances cytotoxicity [32]. To prove enhanced cytotoxicity of CD19-CAR-transduced NK92-cells versus malignant cells, the CAR-NK92 cells’ cytotoxicity was compared to wildtype NK92-cells (wtNK92). Target cells were Daudi, Karpas422 and Jurkat cell lines, as well as primary B cells from healthy donors. By FACS analysis, successful expression of the CAR was verified (Fig. 1A). Also, expression of CD19, FasR and TRAIL receptors (TRAIL-R1 and 2) were tested in the target cells. As expected, Daudi, Karpas422 and primary B cells showed high levels of CD19 (> 97%), whereas Jurkat cells were CD19-negative. (Fig. 1B). FasR and TRAIL-R1/2 were expressed by all target cells (Supp. Fig. 1). To further evaluate susceptibility or resistance towards death-receptor signaling, the target cells’ susceptibility to recombinant FasL (rFasL)-induced apoptosis was evaluated. In these experiments, Karpas422 cells were resistant, whereas apoptosis could be induced in Daudi cells and primary B cells by rFasL (Supp. Fig. 2). Lysis of CD19^+^ target cells was significantly enhanced by insertion of the CD19-CAR into NK92-cells (Fig. 1C). With an Effector:Target-cell (E:T) ratio of 1:1, lysis of Daudi cells increased from 21 to 96% and lysis of Karpas422 cells from 9 to 55% (both *p* < 0.0001). Lysis of primary B cells was 28% and 50%, respectively (*p* = 0.014, Fig. 1C). Jurkat cells (CD19^−^) were equally lysed by wildtype (72%) and CD19-CAR-NK92 cells (71%) (Fig. 1C). These results confirm the CD19-dependent enhanced cytotoxicity of the CD19-CAR-NK92 cells against CD19^+^ target cells.

### Knockout models of CD19-CAR-NK92 cells

**Fig. 2:**
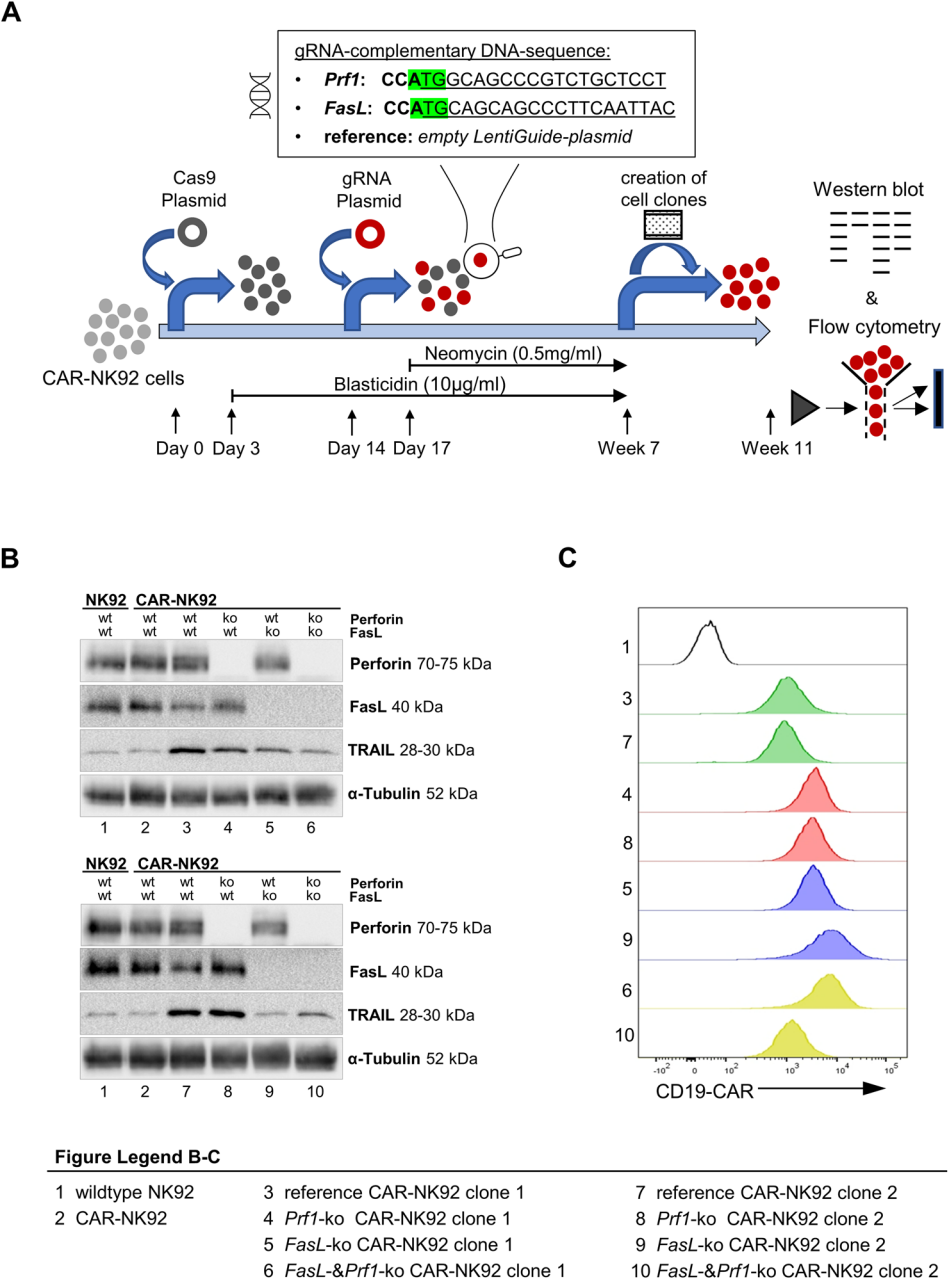
Knockout CD19-CAR-NK92 cell clones. **A** Graphic abstract of the generation of knockout cell clones. CD19-CAR-NK92 cells were initially transduced with Cas9-coding plasmid (day 0). After Blasticidin selection (day 3–14), gRNA-coding plasmids (for *FasL*- or *Prf1*-binding or empty plasmid (reference)) were delivered in Cas9-equipped cells (day 14), followed by Neomycin selection. Creation of the single-cell clones begun after Blasticidin and Neomycin selection for 5 weeks (week 7). Single-cell clones were analyzed for success of knockouts after expanding for at least 4 weeks (week 11). The gRNA-complementary DNA sequences are shown in the box (underlined), the start codon is marked green. **B** Western blotting of perforin-, FasL-, TRAIL- and *α*-Tubulin-expression of the created knockout cell clones (*3–10*). For each knockout experiment, two single-cell clones were analyzed. Corresponding pairs of the cell clones are pictured one below the other (3/7; 4/8; 5/9; 6/10). Wildtype NK92 (*1*) and CD19-CAR-NK92 cells (*2*) were used as relation cells. **C** Histograms of CD19-CAR expression of the knockout cell clones. The corresponding pairs are indicated in the same color (green: reference clones, red: *Prf1*-ko clones, blue: *FasL*-ko clones, yellow: double-ko clones)

Cytotoxicity of NK92-cells is mainly mediated by the perforin/granzyme pathway and the death-receptor pathway [20]. Loss of either pathway should abrogate cytotoxicity of NK92 cells. Knockout models of CD19-CAR-NK92 cells (*Prf1*-ko, *FasL*-ko and double-ko) were generated using CRISPR/Cas9, as both proteins are fundamental for the respective pathway [20] (Fig. 2A). The success of CRISPR/Cas9-mediated knockout was demonstrated by loss of target protein expression via western blot (Fig. 2B).

The overall FasL and Prf1 expression of the single-cell clones was similar compared to wildtype cells. However, TRAIL expression was inhomogeneous. Especially the reference single-cell clones and those with *Prf1*-ko showed increased TRAIL expression levels, compared to wildtype NK92-cells (Fig. 2B). Stable CD19-CAR expression was verified by FACS analysis in all knockout models (Fig. 2C). Subsequently, cytotoxicity of the knockout models was tested to unravel the impact of the respective cytotoxic pathway.

### Cytotoxic activity of CD19-CAR-NK92 knockout models

The cytotoxic activity of the respective knockout models was evaluated in comparison to CD19-CAR-NK92 cells with preserved perforin- and FasL-expression, which served as reference effector cells. To minimize single-cell clone driven bias, we used two cell clones for each experiment and merged the results. Detailed information about the single-cell clones’ cytotoxicity are provided in the supplemental material (Supp. Fig. 3).

**Fig. 3:**
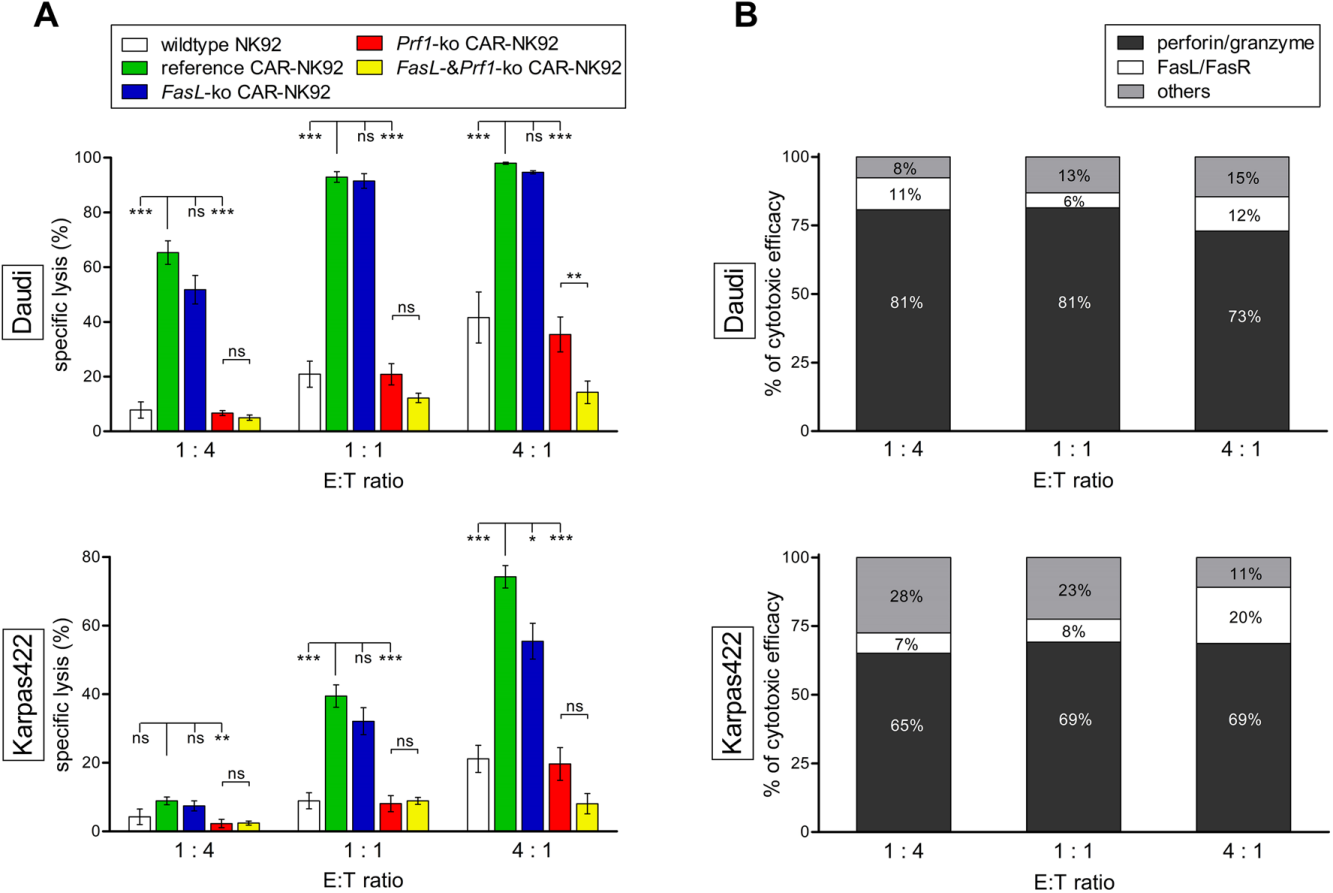
Cytotoxic activity of knockout CD19-CAR-NK92 cells versus CD19^+^ target cells. **A** Specific lysis (%) of Daudi (top) and Karpas422 cells (bottom) by wildtype (white), reference (*green*), *FasL*-ko (*blue*), *Prf1*-ko (*red*) and simultaneous *FasL*- & *Prf1*-ko CD19-CAR-NK92 cells (*yellow*) at E:T ratios of 1:4, 1:1 and 4:1. Cells were co-incubated for 4 h. Two cell clones of each knockout type were tested in triplicates and results were combined into one data group. Values are expressed as means of multiple determination ± SEM. *P* values were calculated by one-way ANOVA followed by Tukey’s test. **B** Proportion (%) of perforin/granzyme pathway, FasL/FasR pathway and others of the total cytotoxic efficacy of CD19-CAR-NK92 cells against Daudi (top) and Karpas422 cells (bottom) at E:T ratios of 1:4, 1:1 and 4:1. The proportions were calculated as following: % = average reduction in cytotoxicity due to respective knockout/cytotoxicity of reference effector cells. The residual lysis of target cells by double-ko effector cells was used to calculate the influence of other pathways, non-perforin and non-FasL mediated

Targeting Daudi cells, *FasL*-ko CD19-CAR-NK92 cells showed no significant difference in cytotoxic activity, compared to reference CD19-CAR-NK92 cells in every E:T ratio tested (Fig. 3A**,** top). Specific lysis of Daudi cells by *FasL*-ko models was 52%, 91%, and 95% (E:T 1:4, 1:1 and 4:1), and 65% (*p* = 0.05), 93% (*p* = 0.90), and 98% (*p* = 0.90) in the respective experiments using reference CD19-CAR-NK92 cells.

Targeting Karpas422 cells, specific lysis by *FasL*-ko CD19-CAR-NK92 cells was 7% compared to 9% by reference CD19-CAR-NK92 cells at E:T of 1:4 (*p* = 0.78), and 32% compared to 39% at 1:1 (*p* = 0.30). With more effector cells (E:T of 4:1), lysis of Karpas422 cells dropped significantly when *FasL* was deleted (55% vs. 74%, *p* = 0.01) (Fig. 3A bottom).

In contrast to knockout of *FasL*, knockout of *Prf1* led to a significantly reduced cytotoxic activity against both, Daudi and Karpas422 cells, at any E:T ratio. Using *Prf1*-ko CD19-CAR-NK92 cells as effector cells, specific lysis of Daudi cells decreased to 7% compared to 65% by reference CD19-CAR-NK92 cells at an E:T ratio of 1:4, 21% compared to 93% at an E:T of 1:1 and 35% compared to 98% at an E:T of 4:1 (all *p* < 0.001) (Fig. 3A top). Specific lysis of Karpas422 cells decreased to 2% by *Prf1*-ko effector cells compared to 9% by reference effector cells at an E:T ratio of 1:4 (*p* < 0.01), 8% compared to 39% at an E:T of 1:1 (*p* < 0.001) and 20% compared to 74% at an E:T of 4:1 (*p* < 0.001) (Fig. 3A bottom).

Compared to an exclusive *Prf1*-ko, simultaneous knockout of *FasL* and *Prf1* resulted in no additional cytotoxic deficiency at an E:T ratio of 1:4 and 1:1, in both, Daudi and Karpas422 cells (all *p* > 0.05). However, at an E:T ratio of 4:1, the cytotoxic activity was significantly decreased using double-knockout models targeting Daudi cells (14% vs. 35%, *p* < 0.01), whereas a nonsignificant reduction was obvious targeting Karpas422 cells (8% vs. 20%, *p* = 0.24). Overall, the CD19-CAR-NK92 cells’ cytotoxicity was superior to wtNK92-cells (Fig. 3A).

Based on these results, each pathway’s proportion of the total cytotoxic efficacy of CD19-CAR-NK92 cells was calculated (% = average reduction in cytotoxicity due to the respective knockout/cytotoxicity of reference effector cells). Cytotoxicity was largely mediated via the perforin/granzyme pathway (Daudi by 73–81%, Karpas422 cells by 65–69%). The proportion of the FasL/FasR pathway was lower, but increased in higher E:T ratios from 7% (1:4) to 20% (4:1) (Fig. 3B).

Concluding, both pathways mediate cytotoxicity of CAR-NK92 cells, but loss of the perforin/granzyme pathway reduced the cytotoxic activity of CAR-NK92 cells more profound, than loss of the Fas-pathway. Moreover, knockout of *FasL* could be compensated by functional perforine/granzyme pathway in most conditions, which was not the case vice versa.

### Cytotoxic activity of CD19-CAR-NK92 knockout models against primary human B cells

**Fig. 4:**
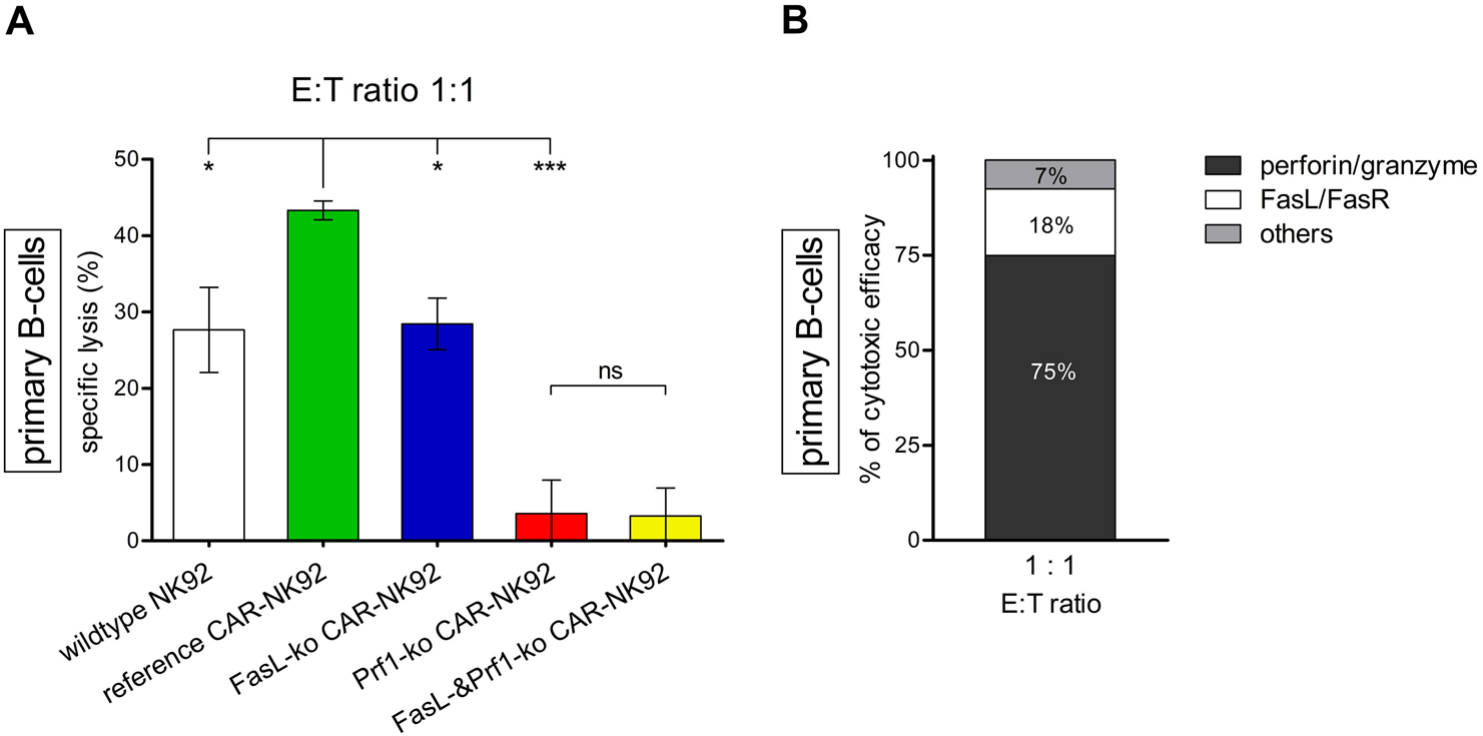
Cytotoxic activity of knockout CD19-CAR-NK92 cells versus human primary B cells. **A** Specific lysis (%) of primary B cells by wildtype (white), reference (*green*), *FasL*-ko (*blue*), *Prf1*-ko (*red*) and *FasL-* & *Prf1*-ko CD19-CAR-NK92 cells (*yellow*) at E:T ratios of 1:1. Cells were co-incubated for 4 h. Primary B cells were isolated from blood of two healthy donors. Again, two clones of each knockout type were tested, and results were combined into one data group. Data values are expressed as means of multiple determination ± SEM. *P* values were calculated by one-way ANOVA followed by Tukey’s test. **B** Proportion (%) of perforin/granzyme pathway, FasL/FasR pathway and others of the total cytotoxic efficacy of CD19-CAR-NK92 cells against primary B cells at E:T ratios of 1:1. Calculation of proportions as described in legend of Fig. 3

To confirm the importance of the perforin/granzyme pathway for the cytotoxic activity of CD19-CAR-NK92 cells, even against target cells with fully functional death-receptor signaling, we used primary human B cells as target cells in a further experiment. At an E:T ratio of 1:1, specific lysis of primary B cells was 28% by *FasL*-ko cells and 43% by reference CD19-CAR-NK92 cells (*p* = 0.04). On the contrary, *Prf1*-ko decreased the cytotoxicity to 4% (*p* < 0.001) (Fig. 4A green vs. blue and red columns). Using double-ko effector cells compared to exclusive *Prf1*-ko effector cells, no additional effect was found (3% vs. 4%; *p* = 0.90) (Fig. 4A yellow and red columns).

The cytotoxic efficacy was similarly mediated against non-malignant cells, as it was in malignant cells (Fig. 4B). Thus, the importance of the perforin/granzyme pathway is not a tumor specific finding.

### Cytotoxic activity and inhibition of TRAIL

**Fig. 5:**
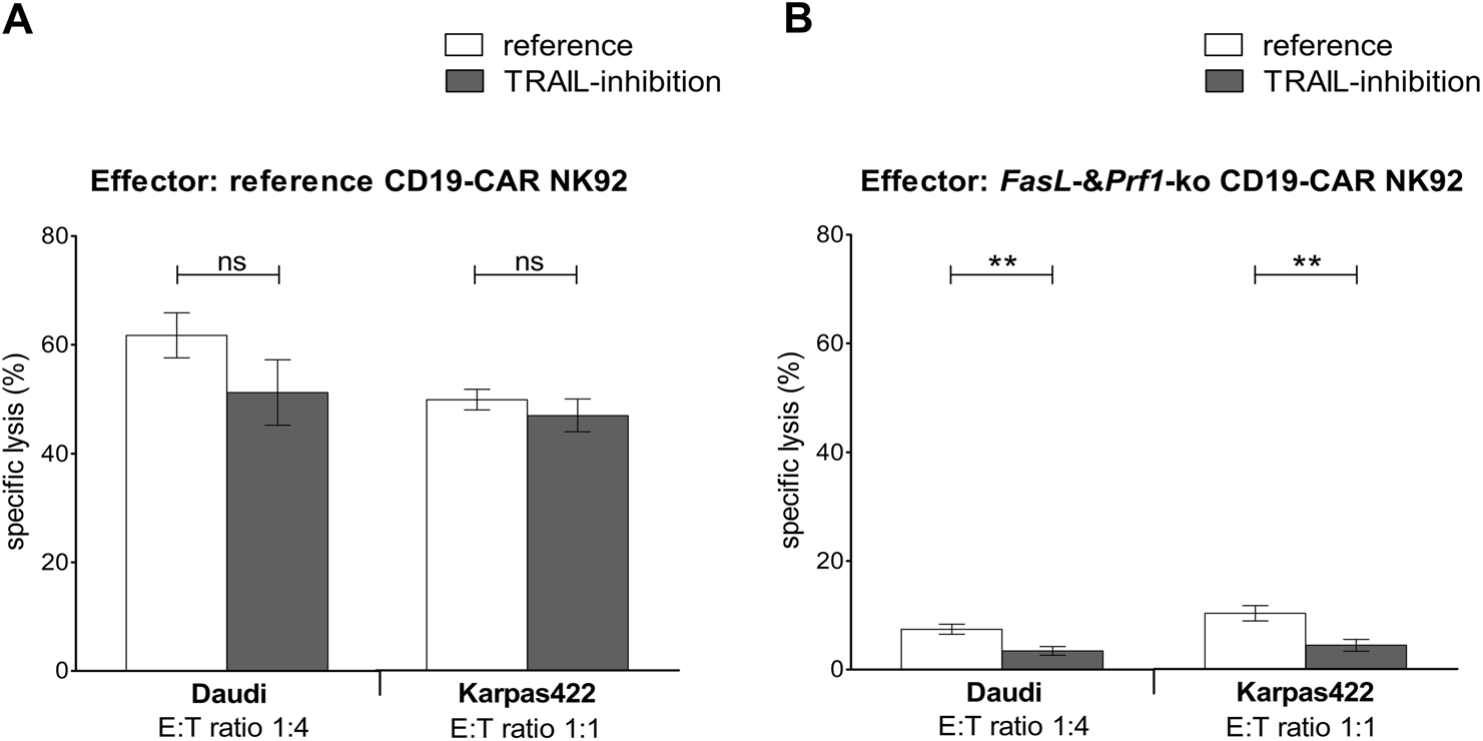
Cytotoxic activity of CD19-CAR-NK92 cells despite inhibition of TRAIL. **A** Specific lysis (%) of Daudi and Karpas422 cells by reference CD19-CAR-NK92 cells with (gray) and without (white) anti-TRAIL antibody (5 μg/ml). **B** Specific lysis (%) of Daudi and Karpas422 cells by *FasL-* & *Prf1*-ko CD19-CAR-NK92 cells with (gray) and without (white) anti-TRAIL antibody (5 μg/ml). **A** + **B** Cells were co-incubated for 4 h. Two cell clones were used, each assay was performed in triplicates and the results were combined in one data group. Values are expressed as means of multiple determination ± SEM. *P* values were calculated by unpaired *t* test

Since double-knockout effector cells were still capable of killing CD19^+^ target cells, we additionally investigated the impact of TRAIL-mediated cytotoxicity using an anti-TRAIL antibody for specific inhibition [31]. By that we expected further reduced cytotoxic activity, especially when double-knockout models were used as effector cells. An E:T ratio of 1:4 was used when Daudi cells were targeted, because of the high specific lysis in the other E:T ratios and a potential risk of immeasurable effects of a TRAIL-blockade. Experiments with Karpas422 cells were performed at an E:T ratio of 1:1. The application of the anti-TRAIL antibody did not significantly reduce the cytotoxic activity of reference CD19-CAR-NK92 models, although these cells showed higher TRAIL expression in western blots. Specific lysis of Daudi cells by CD19-CAR-NK92-cells was 51% in the presence of the anti-TRAIL antibody and 62% without (*p* = 0.18). Lysis of Karpas422 cells was 47% in the presence of the anti-TRAIL antibody and 50% without (*p* = 0.43) (Fig. 5A). However, using the double-knockout effector cells, the residual specific lysis of the target cells could be further reduced by inhibition of TRAIL. In Daudi cells, specific lysis decreased to 3% in the presence of the anti-TRAIL antibody, compared to 7%. In Karpas422 cells, the specific lysis decreased to 4% compared to 10% (both *p* < 0.01) (Fig. 5B). These results point out that inhibition of TRAIL-mediated cytotoxicity can be compensated by other pathways.

## Discussion

In this study, *Prf1*, *FasL*, and double-knockout models of CD19-CAR-NK92 cells were generated by a CRISPR-Cas9 system, to investigate cytotoxic mechanisms. To the best of our knowledge, this is the first study of *Prf1* and *FasL-*ko models in CAR-NK92 cells. The study’s objective was to analyze the major cytotoxic pathways and their respective contribution in mediating cytotoxicity.

The benefits of NK-cells—and particularly of the NK92-cell line—as effector cells in CAR-nant cells are able to avoid NK-cell activation and subsequent killing by several immunoescape mechanisms [21, 33]. CARs have the ability to overcome this resistance, and enhance or restore the cytotoxicity [11, 18, 34]. The optimal CAR-design in NK-cells, particularly the co-stimulatory domain, is a matter of debate [12, 13, 34]. Some preclinical and clinical trials tested second-generation CAR constructs in NK92 cells with different co-stimulatory domains and antigen binding sites, showing substantial anti-tumor efficacy [15]. In our study, insertion of the 4-1BB CD19-CAR leads to enhanced cytotoxicity against CD19^+^ target cells, especially against lymphoma cells, which were partly resistant toward wtNK92-cells.

The cytotoxic activity of NK92-cells is largely dependent on two pathways: the perforin/granzyme pathway and the death-receptor pathway, the latter being activated by FasL or TRAIL [19, 20, 35]. As previously described, knockout of *Prf1* leads to abrogation of apoptosis induction via the perforin/granzyme pathway [36, 37]. Likewise, *FasL* is the only gene encoding for the FasL, by which the Fas-dependent pathway is activated [20, 38]. We generated knockout models of the respective cytotoxic pathway’s gatekeeper proteins. Generating single-cell clones may bias results, but it guaranteed complete knockout of the target genes. To reduce the potential bias, two single-cell clones of each knockout model were used for every experiment.

The findings of our study indicate the perforin/granzyme pathway as the dominant contributor of CAR-NK92 cells’ cytotoxic activity. This was irrespective of the target cells’ susceptibility to death-receptor signaling. Fas-signaling could not compensate *Prf1* knockout, even in cells susceptible to rFasL (Daudi and primary B cells). Loss of the perforin/granzyme pathway strongly impaired cytotoxicity in both tested cell lines and primary B cells, in any E:T ratio. These findings are supported by previous studies, which revealed the perforine/granzyme pathway as essential for anti-tumor efficacy of wtNK-cells [39, 40].

Another finding in this study is the growing influence of the Fas-pathway in conditions with higher E:T ratios. This was shown in single-knockout and double-knockout experiments. Only with higher E:T ratios, *Prf1-*ko CAR-NK92 cells were able to kill a substantial proportion of target cells. Double-knockout of *Prf1* and *FasL* had an additive effect, but only in higher E:T ratios. This is of particular interest, since NK92-cells are used irradiated in clinical trials, which limits the effector cell count by abrogation of substantial proliferation. Thus, low E:T ratios at the tumor site can be expected in vivo. Fas-related antigen independent killing via cell–cell interactions has been described for CAR-T cells, and time to effector/target-cell interaction and time to target-cell death can be shortened by increasing effector cell counts [37, 41]. This provides a potential explanation for the higher impact of Fas-dependent killing in higher E:T ratios.

Previous studies revealed the death-receptor pathway as the slower pathway (compared to perforine/granzyme) to induce apoptosis in target cells, which may bias experiments [20]. In our study, co-incubation of effector and tumor cells was 4 h. This widely excludes the possibility of missing FasL-expression due to insufficient co-incubation time as a cause for observed limited importance for cytotoxic action [20, 37]. This is underlined by the significant impact of the FasL/FasR pathway in higher E:T ratios.

The study’s next finding is the limited impact of TRAIL, if other cytotoxic pathways are functional. Inhibition of TRAIL in CAR-NK92 cells with preserved *Prf1* and *FasL* function did not impair cytotoxicity. In double-knockout CAR-NK92 cells (*Prf1* and *FasL*)*,* inhibition of TRAIL nearly abrogated the residual cytotoxic effects completely. The Fas- and TRAIL-pathway both activate death-receptor signaling and caspase-dependent apoptosis in the target cell, showing redundant characteristics [20]. This may explain both, the low contribution of TRAIL to cytotoxicity in CAR-NK92 cells with preserved FasL function, but the significant impact in double-knockout models.

For unknown reasons, Daudi cells were more susceptible to CAR-NK92-mediated killing compared to Karpas422. Target expression (CD19) was not different, and FasR/TRAIL receptors were expressed by both cells. Moreover, FasL-signaling had only a marginal influence in Daudi cells in the respective knockout experiments. This makes it unlikely that the susceptibility of Daudi cells to Fas-induced apoptosis plays a role, rather suggesting other cell line specific immunoescape features of Karpas422, impairing cytotoxicity. However, the results are in-line with previous studies showing inhomogeneous susceptibility of different cell lines to CAR-NK92 cells [18, 42].

Our study has several implications for further clinical and preclinical research. Since the dominant impact of the perforin/granzyme pathway on cytotoxicity, it seems reasonable to modulate target cells’ susceptibility to cytotoxic granules by, e.g., anti-SerpinB9 therapy, to further enhance CAR-NK92 cells efficacy [43]. Since wtNK-cells switch from the perforine/granzyme pathway to the Fas-pathway during serial killing, the CAR’s impact on serial killing and the recruitment on dormant NK-cells is another interesting field for future studies [37, 44]. A recent study revealed downregulation of genes involved in Fas-mediated apoptosis as an important feature of primary resistance against CAR-T cells in B-ALL [10]. Based on our findings, this mechanism of resistance may be overcome by CAR-NK92-cells. In lymphoma, alterations in death-receptor signaling are frequent, yet with unknown implications for therapy and prognosis [45, 46]. Their contribution to resistance against CAR-therapies is of special interest in CD19-CAR-therapy [47, 48]. Both cell lines used in our study have known alterations in the Fas-pathway. A benefit of CAR-effector cells relying on the perforin/granzyme pathway, rather than the Fas-pathway, may be confirmed in future trials.

The limitations of our study are the missing in vivo experiments since the kinetics and cytotoxic pathways may be influenced by a different cytokine milieu and tumor microenvironment in vivo. Moreover, the CAR design may have impact on the respective cytotoxic pathways used by effector cells, thus our findings cannot be transferred to other CAR-constructs [34]. Furthermore, it remains unclear if our findings are transferable to NK-cells in general.

In summary, our study proves great dependency of 4-1BB-CD19-CAR-NK92 cells on the perforin/granzyme pathway to kill target cells.

### Supplementary Information

Below is the link to the electronic supplementary material.

Supplementary file1 (DOCX 1215 KB)
