# Supplementary material for: Cytotoxicity of CD19-CAR-NK92 cells is primarily mediated via perforin/granzyme pathway
Source: Cancer Immunol Immunother. 2023 Apr 13;72(8):2573–83. doi: 10.1007/s00262-023-03443-1 (PMC10361870; doi:10.1007/s00262-023-03443-1)
Supplement: Supplementary file 1 — Supplementary file1 (DOCX 1215 KB) [file 262_2023_3443_MOESM1_ESM.docx]

**Supplemental Material**

**Supplement Figure 1**


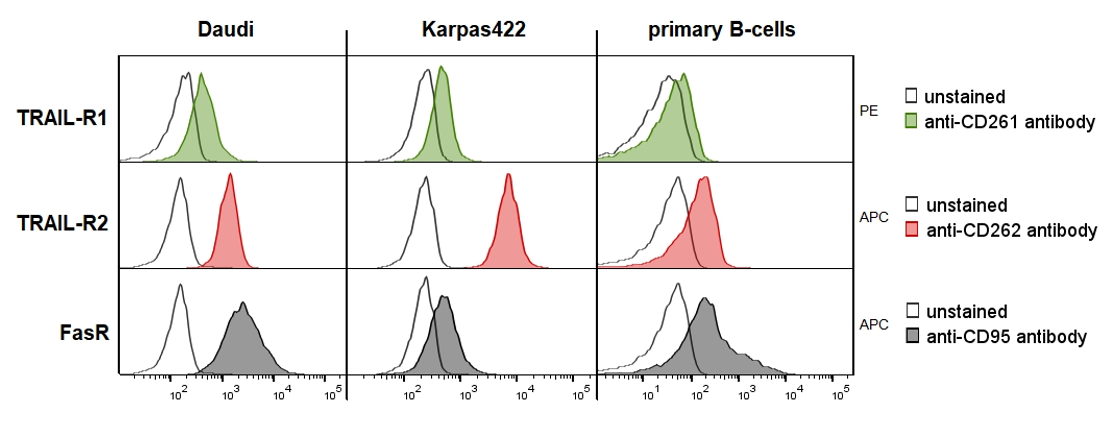


**Supp. Fig. 1: Target cells’ expression of TRAIL-Receptors 1 and 2 (TRAIL-R1/2) and expression of Fas-Receptor (FasR).** All different target cells expressed the respective receptor. TRAIL-R1 was expressed in lower levels, compared to TRAIL-R2. Expression of Fas-R differed between the target cells and was the highest in Daudi cells, and similar in Karpas422 and primary B-cells.

**Supplement Figure 2**





***Supp. Fig. 2: Cell viability assay in presence of recombinant human Fas-L (rFasL).*** The susceptibility of target cells (Daudi, Karpas422, Jurkat and primary B-cells) to the Fas pathway was evaluated using recombinant FasL (Cell Signaling Technology). For this purpose, cells were seeded on a 96-well plate in 100µl medium at a cell count of 30,000 cells/well in case of the cell lines and 100,000 cells/well in case of primary B-cells. To ensure sufficient cross-linking between FasL molecules, the cells were treated in the presence of 10 µg/ml anti-His antibody (R&D Systems). Cells were incubated for 24h. The viability after treatment was assessed using the Colorimetric Cell Viability Kit III (XTT; PromoKine) according to the manufacturer’s protocol and determining the OD_450_ - OD_630_. Primary B-cells were stimulated with 300ng/ml BAFF and 30 ng/ml IL-4 during FasL-treatment to achieve sufficient metabolic activity for the XTT-assay. The Fas-sensitive cell line Jurkat served as positive control.

Primary B-cells and Daudi cells were susceptible towards rFasL, whereas Karpas422 showed a non-significant trend towards lower viability.

**Supplement Figure 3**


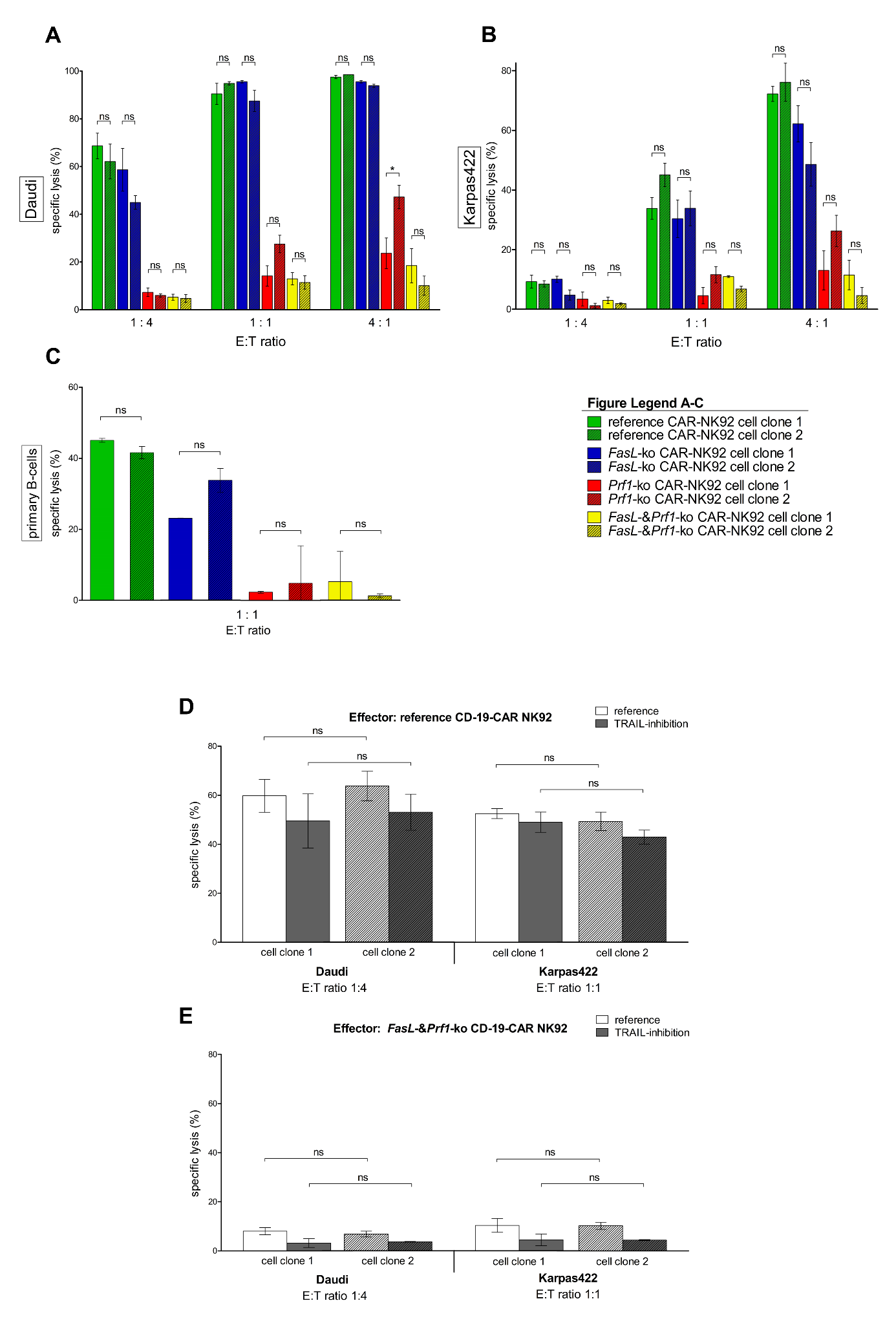


**Supp. Fig. 3:** **Single-cell clones’ cytotoxicity of generated knockout models.** The results of the two single-cell clones are merged in Figure 3. A: Cytotoxicity of the respective single-cell clone in killing-assays targeting Daudi cells. B: Cytotoxicity of the respective singe-cell clones targeting Karpas422. C: Cytotoxicity of the respective single-cell clones targeting primary B-cells. D: Cytotoxicity of CD19-CAR-NK92 cells with preserved FasL and Prf1 expression targeting Daudi (left) and Karpas422 (right) in presence of a TRAIL-inhibitor. E: Cytotoxicity of double-knockout models (Prf1 + FasL) targeting Daudi (left) and Karpas422 (right) in presence of a TRAIL-inhibitor. Concluding, no significant pattern of different cytotoxicity could be seen between the single-cell clones of each knockout model. This makes a bias regarding the cytotoxicity of the models caused by single-cell generation less likely.
